# Glycerine and Carbolic Acid for Burns

**Published:** 1870-01

**Authors:** T. Williams


### GLYCERINE AND CARBOLIC ACID FOR BURNS.

*To the Editors of* THE CHICAGO MEDICAL JOURNAL:

AT the Gaiety Theatre fire in this city, some three weeks since, Mr. Bangs, photographer, was badly burned about the face and hands. The latter were affected so that the skin peeled off like gloves. As soon afterwards as possible, the burns were dressed with linseed oil, and morphia was administered to relieve pain. I first saw the patient next morning. I dressed the hands and face with the following:

℞ Glycerine, - - - - - - ℥ vj;
Acidi Carbol., - - - - - - ʒ j; M.

This augmented the pain considerably for an hour or more. It was applied freely, with a camel's hair pencil, and the fingers afterwards wrapped up, separately, with strips of linen lint. The dressing was changed daily, by soaking the lint in warm water, washing the parts by squeezing warm soap-suds out of a sponge over them, and re-applying the glycerine and carbolic acid. The patient had to have morphine once or twice daily, to relieve the pain. Eighteen days after, while others burnt no worse, but had received different treatment, were still suffering, Mr. Bang's wounds had healed and new skin formed, leaving no scar on the face, although the epidermis had all peeled off. After this, simple cerate, or pure glycerine, was substituted, and he is now able to resume his work, although his hands are still very tender.

In the October number of the *American Journal of the Medical Sciences*, is a statement of a severe burn of the legs, from the knees down, treated by C. C. Lange, M.D., of Pittsburg, by linseed oil, 8 parts, carbolic acid, 1 part. I would say to the profession that this is too strong, too large a proportion of the carbolic acid, and must have produced intense suffering, besides the danger incurred by its *absorption* from so large a surface. A drachm to four ounces of glycerine, is as strong as should be applied, or as is necessary. I consider the glycerine preferable to the oil — is mild, protects from the air, and mixes thoroughly with the acid, which it dissolves.

On a portion of one hand, at the patient's request, and on his face, I tried the application of furniture *varnish*, in which he had great faith, from seeing a paragraph in the papers recommending it. The parts did not do so well, apparently, and it was discontinued, and the glycerine resumed, when they satisfactorily healed. T. WILLIAMS, M.D.

